# Drinking Ice-Cold Water Reduces the Severity of Anticancer Drug-Induced Taste Dysfunction in Mice

**DOI:** 10.3390/ijms21238958

**Published:** 2020-11-25

**Authors:** Ayana Osaki, Keisuke Sanematsu, Junichi Yamazoe, Fumie Hirose, Yu Watanabe, Yuko Kawabata, Asami Oike, Ayaka Hirayama, Yu Yamada, Shusuke Iwata, Shingo Takai, Naohisa Wada, Noriatsu Shigemura

**Affiliations:** 1 Section of Oral Neuroscience, Graduate School of Dental Science, Kyushu University, 3-1-1 Maidashi, Higashi-ku, Fukuoka 812-8582, Japan; a-osaki@dent.kyushu-u.ac.jp (A.O.); f-hirose@dent.kyushu-u.ac.jp (F.H.); yu-w-23@dent.kyushu-u.ac.jp (Y.W.); ykawabata@dent.kyushu-u.ac.jp (Y.K.); miyake.asami.638@s.kyushu-u.ac.jp (A.O.); hirayama.ayaka.634@s.kyushu-u.ac.jp (A.H.); yamada.yu.540@s.kyushu-u.ac.jp (Y.Y.); ganchan@dent.kyushu-u.ac.jp (S.I.); takashin@dent.kyushu-u.ac.jp (S.T.); 2 Division of General Dentistry, Kyushu University Hospital, Kyushu University, 3-1-1 Maidashi, Higashi-ku, Fukuoka 812-8582, Japan; wada@dent.kyushu-u.ac.jp; 3 Oral Health/Brain Health/Total Health Research Center, Graduate School of Dental Science, Kyushu University, 3-1-1 Maidashi, Higashi-ku, Fukuoka 812-8582, Japan; 4 Research and Development Center for Five-Sense Devices, Kyushu University, 744 Motooka, Nishi-ku, Fukuoka 819-0395, Japan; 5 Section of Oral Healthcare and Dentistry Cooperation, Division of Maxillofacial Diagnostic and Surgical Science, Faculty of Dental Science, Kyushu University, 3-1-1 Maidashi, Higashi-ku, Fukuoka 812-8582, Japan; yamazoe@dent.kyushu-u.ac.jp

**Keywords:** taste, taste disorder, docetaxel, cisplatin, 5-fluorouracil

## Abstract

Taste disorders are common adverse effects of cancer chemotherapy that can reduce quality of life and impair nutritional status. However, the molecular mechanisms underlying chemotherapy-induced taste disorders remain largely unknown. Furthermore, there are no effective preventive measures for chemotherapy-induced taste disorders. We investigated the effects of a combination of three anticancer drugs (TPF: docetaxel, cisplatin and 5-fluorouracil) on the structure and function of mouse taste tissues and examined whether the drinking of ice-cold water after TPF administration would attenuate these effects. TPF administration significantly increased the number of cells expressing apoptotic and proliferative markers. Furthermore, TPF administration significantly reduced the number of cells expressing taste cell markers and the magnitudes of the responses of taste nerves to tastants. The above results suggest that anticancer drug-induced taste dysfunction may be due to a reduction in the number of taste cells expressing taste-related molecules. The suppressive effects of TPF on taste cell marker expression and taste perception were reduced by the drinking of ice-cold water. We speculate that oral cryotherapy with an ice cube might be useful for prophylaxis against anticancer drug-induced taste disorders in humans.

## 1. Introduction

Combination chemotherapy is widely used to treat patients with cancer [1]. Agents used for combination chemotherapy include docetaxel, a plant alkaloid that inhibits cell division and replication by stabilizing microtubule assembly, cisplatin, an alkylating agent that interferes with the transcription of DNA into RNA, and 5-fluorouracil (5-FU), a pyrimidine analog that inhibits DNA and RNA synthesis by misincorporation of its metabolites into RNA and DNA. These anticancer agents are thought to exert additive or synergistic effects to trigger programmed cell death (apoptosis) in rapidly proliferating tumor cells. For example, patients with head and neck squamous cell carcinoma who received TPF (docetaxel, cisplatin and 5-fluorouracil) chemotherapy followed by chemoradiotherapy showed significantly longer survival than patients who received the same treatment without docetaxel [2].

However, 50–70% of patients experience taste disorders during treatment with anticancer agents [3,4,5,6,7]. Chemotherapy-induced taste disorders are associated with lower quality of life (QOL), reduced ability to eat and drink (anorexia), poorer nutritional status and a decline in activities of daily living (ADL) [8]. Currently, there are no effective measures to prevent anticancer drug-induced taste disorders, and the development of new treatment strategies will require a better understanding of the factors underlying this adverse effect of chemotherapy. Anticancer drugs can affect mature taste cells as well as the proliferation and differentiation of taste stem/progenitor cells [9], and cytotoxic damage to these cell types may be a cause of taste disorders.

Taste is transduced by taste receptor cells located in taste buds on the anterior and posterior tongue surfaces as well as the soft palate, oropharynx and larynx. Recent molecular studies have discovered candidate receptors for five basic tastes: taste receptor type 1 member (T1R) 2 and T1R3 heterodimers (G protein-coupled receptors [GPCRs]) for sweet; T1R1 and T1R3 heterodimers (GPCRs) for umami; taste receptor type 2 (T2R, a GPCR) for bitter; epithelial sodium channel (ENaC) for salty; and otopetrin-1 (Otop1, a proton channel) for sour [10,11]. Each taste receptor is expressed in a distinct subset of cells within the taste buds, suggesting that the discrimination of taste quality occurs at the most peripheral taste cells. Activation of taste-specific receptors leads to the mobilization of downstream signaling pathways such as Gα-gustducin, phospholipase C-beta 2 (PLCβ2) and transient receptor potential cation channel M5 (Trpm5), which are recruited by the GPCRs. Activation of these pathways in taste cells generates action potentials that stimulate the release of neurotransmitters onto afferent nerve fibers, which convey the signal to the brainstem. Notably, taste cells regenerate continuously with an average turnover of 5–20 days [10,12]. Furthermore, each type of taste cell appears to have a distinct life span. Cells that discriminate taste via GPCRs (sweet, bitter and umami) have a half-life of about 8 days, while those that detect taste via channels (sour) have a half-life of about 22 days, although a subset may persist for much longer [13]. However, little is known about the effects of anticancer agents on the different types of taste cell.

Oral cryotherapy, which involves cooling the mouth with ice chips, is a low-cost and safe treatment that lacks serious side effects and is generally well tolerated by patients [13]. Oral cryotherapy is thought to help prevent oral mucositis in patients receiving bolus 5-FU or high-dose melphalan chemotherapy by narrowing oral blood vessels and thereby reducing local exposure to anticancer agents. For example, oral cryotherapy given at the time of chemotherapy was shown to significantly reduce 5-FU-associated mucositis [14]. The above finding raises the possibility that oral cryotherapy might also be effective at reducing the adverse effects of anticancer agents on taste cells.

We hypothesized that anticancer drugs might induce taste disorders in a taste cell type-specific manner, with fast-cycling cells (sweet, bitter and umami) being more susceptible to injury than slow-cycling cells (salty and sour). In addition, we conjectured that the drinking of ice-cold water after anticancer drug administration might inhibit the development of drug-induced taste disorders. To explore these hypotheses, we investigated the effects of anticancer drugs (TPF) on the expressions of taste cell markers (T1R3 for sweet, gustducin for sweet/bitter, PLCβ2 for sweet/bitter/umami and carbonic anhydrase IV [CaIV] for sour), apoptosis markers and proliferation markers in the taste tissues of mice. We further evaluated the effects of TPF on taste nerve responses in mice. Additionally, we determined whether the drinking of ice-cold water inhibited the actions of TPF.

## 2. Results

### 2.1. Intraperitoneal Injection of Anticancer Drugs (TPF) Did Not Affect Mouse Body Weight

**Figure 1:**
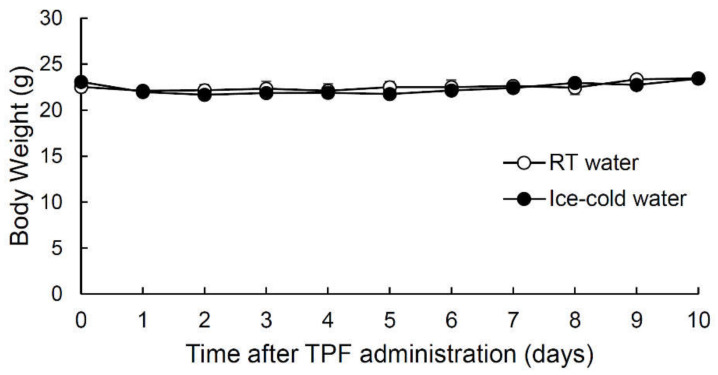
Temporal changes in the body weight of mice that drank either room temperature (RT) water or ice-cold water during the 10-day period following the intraperitoneal administration of TPF (docetaxel, cisplatin and 5-fluorouracil). Data are expressed as the mean ± SEM (*n* = 4 mice, each).

Patients undergoing chemotherapy were reported to exhibit a significant reduction in body weight [15]. Therefore, we monitored the time-course of the changes in the body weight of mice that drank room temperature (RT) water or ice-cold water after a single intraperitoneal injection of TPF (20 mg/kg docetaxel, 6 mg/kg cisplatin and 17 mg/kg 5-FU) (Figure 1, Supplementary Table S1). There was no significant change in body weight during the 10 days following TPF administration for both mice that drank RT water and those that drank ice-cold water (all *p*-values > 0.05, one-way analysis of variance (ANOVA) and Tukey’s post-hoc test). Furthermore, there were no significant differences in body weight between mice that drank RT water after TPF administration and those that drank ice-cold water (*F*_1,60_ = 0.34, *p* > 0.05, two-way repeated-measures ANOVA, effect of drinking condition; Figure 1, Supplementary Table S1). The above results indicate that the administration of TPF at the drug concentrations used did not cause severe injury to the mice. Therefore, we used this administration regimen for all subsequent experiments.

### 2.2. Taste Cell Apoptosis after TPF Administration Was Suppressed by the Drinking of Ice-Cold Water

**Figure 2:**
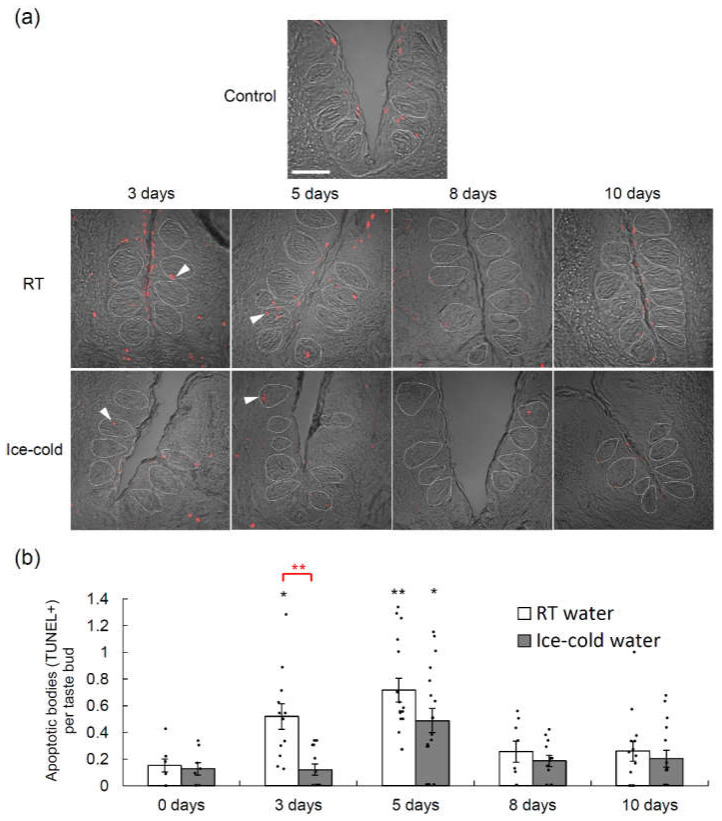
Terminal deoxynucleotidyl transferase-mediated dUTP nick end labeling (TUNEL) assays performed in the circumvallate papillae of mice that drank room temperature (RT) water or ice-cold water during the 10-day period following the intraperitoneal administration of TPF (docetaxel, cisplatin and 5-fluorouracil). (**a**) TUNEL signals (red) in the circumvallate papillae of mice that drank RT water or ice-cold water before (0 days) and 3, 5, 8 and 10 days after TPF administration. Arrowheads indicate TUNEL-positive apoptotic bodies. Dotted lines outline individual taste buds. Scale bar: 50 μm. (**b**) Quantitation of the number of TUNEL-positive cells per taste bud at different time points after TPF administration for mice that drank RT water and those that drank ice-cold water. Data are expressed as the mean ± SEM (*n* = 8–16 trenches each). Dots indicate individual data points. * (in black) *p* < 0.05 vs. control (0 days), ** (in black) *p* < 0.01 vs. control (0 days) (one-way analysis of variance (ANOVA) and Tukey’s post-hoc test); ** (in red) *p* < 0.01 for comparison between RT water and ice-cold water (two-way ANOVA and post-hoc *t*-test).

Each of the anticancer drugs in TPF induces apoptotic cell death [16,17,18]. Therefore, we used the terminal deoxynucleotidyl transferase-mediated dUTP nick end labeling (TUNEL) assay to determine whether TPF induced the apoptosis of taste cells (Figure 2, Supplementary Tables S1 and S2). For these experiments (and our subsequent anatomical studies), we focused on the circumvallate papillae (CVP), which are large structures located in the posterior part of the tongue that have deep epithelial trenches containing ~150 taste buds bilaterally. The TUNEL signals in the taste buds of the CVP increased significantly after TPF administration in both mice that drank RT water and those that drank ice-cold water, and the peak TUNEL signal was observed 5 days after TPF administration in both groups (*p* < 0.05 vs. control, one-way ANOVA and Tukey’s post-hoc test; Figure 2, Table S1). However, the TUNEL signal was significantly lower in mice that drank ice-cold water than in mice that drank RT water (*F*_1,115_ = 11.62, *p* < 0.001, two-way ANOVA, effect of drinking condition; Figure 2, Supplementary Tables S1 and S2).

### 2.3. Taste Cell Proliferation after TPF Administration Was Suppressed by the Drinking of Ice-Cold Water

**Figure 3:**
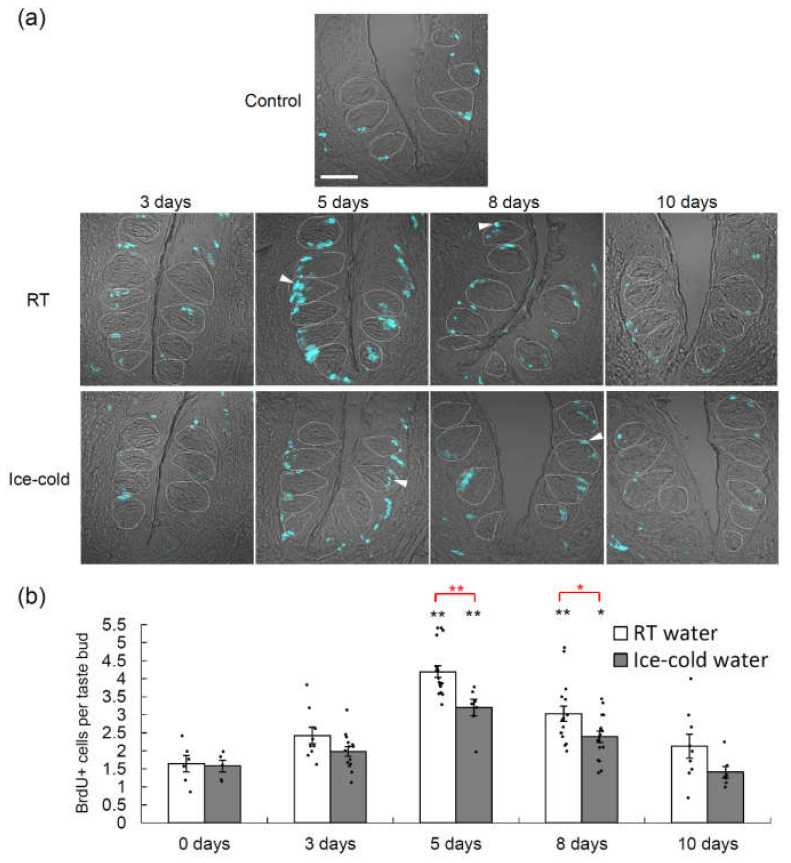
Bromodeoxyuridine (BrdU) labeling of the circumvallate papillae of mice that drank room temperature (RT) water or ice-cold water during the 10-day period following the administration of TPF (docetaxel, cisplatin and 5-fluorouracil). (**a**) BrdU signals (cyan) in the circumvallate papillae before (0 days) and 3, 5, 8 and 10 days after TPF administration for mice that drank RT water or ice-cold water. Arrowheads indicate BrdU-positive proliferative cells. Dotted lines outline individual taste buds. Scale bar: 50 μm. (**b**) Quantitation of the number of BrdU-positive cells per taste bud at different time points after TPF administration for mice that drank RT water and those that drank ice-cold water. Data are expressed as the mean ± SEM (*n* = 5–18 trenches, each). Dots indicate individual data points. * (in black) *p* < 0.05 vs. control (0 days), ** (in black) *p* < 0.01 vs. control (0 days) (one-way ANOVA and Tukey’s post-hoc test); * (in red) *p* < 0.05, ** (in red) *p* < 0.01 for comparison between RT water and ice-cold water (two-way ANOVA and post-hoc *t*-test).

Bromodeoxyuridine (BrdU) is a thymidine analogue that is incorporated into newly synthesized DNA during the S-phase of the cell cycle and thus can be used to identify proliferating cells [19,20]. We performed BrdU-labeling experiments to evaluate taste cell proliferation at different time points after TPF administration (Figure 3, Supplementary Tables S1 and S3). For both groups of mice (those that drank RT water and those that drank ice-cold water), the BrdU signals in the taste buds of the CVP increased significantly after TPF administration and peaked at 5 days (*p* < 0.05 vs. control at days 5 and 8 for both groups, one-way ANOVA and Tukey’s post-hoc test; Figure 3, Supplementary Table S1) before declining to basal (control) levels. Furthermore, there was a significant difference in the number of cells expressing BrdU signals between mice that drank RT water and those that drank ice-cold water (*F*_1,97_ = 41.59, *p* < 0.001, two-way ANOVA, effect of drinking condition; Figure 3, Supplementary Table S1).

### 2.4. TPF-Induced Reductions in the Numbers of Cells Expressing Various Taste Cell Markers Were Suppressed by the Drinking of Ice-Cold Water

**Figure 4:**
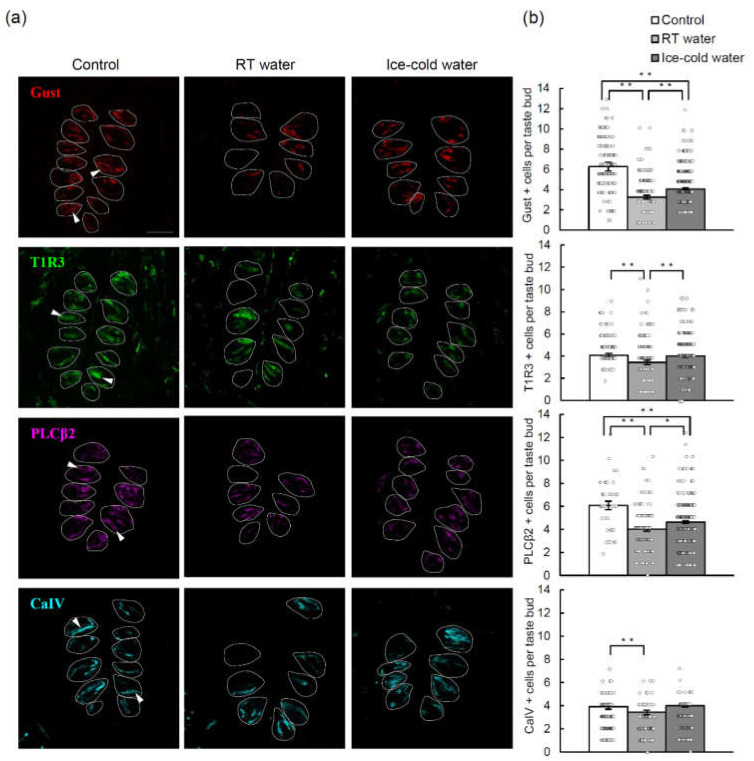
The number of taste bud cells expressing taste cell markers in mice that drank room temperature (RT) water or ice-cold water for 10 days following the administration of TPF (docetaxel, cisplatin and 5-fluorouracil). (**a**) Expression of gustducin (Gust, a G-protein mediating bitter and sweet taste transduction; red), taste receptor type 1 member 3 (T1R3, a sweet or umami receptor component; green), phospholipase C-beta 2 (PLCβ2, a bitter/sweet/umami transduction molecule; magenta) and carbonic anhydrase-4 (CaIV, a sour taste-sensitive cell marker; cyan) in the circumvallate papillae before (control) and 10 days after TPF administration in mice that drank RT water or ice-cold water. Arrows indicate marker-positive cells. Dotted lines outline individual taste buds. Scale bar: 50 μm. (**b**) Quantitation of the number of immunoreactive cells per taste bud. Data are expressed as the mean ± SEM (*n* = 100–286 taste buds, each). Dots indicate individual data points. * *p* < 0.05, ** *p* < 0.01 (one-way ANOVA and Tukey-Kramer post-hoc test).

Next, we performed immunohistochemical analyses to investigate whether the number of mouse taste bud cells in the CVP was altered by TPF administration and whether any such effects were suppressed by the drinking of ice-cold water (Figure 4, Supplementary Tables S4 and S5). Taking into consideration the timing of the peak effect of a single TPF injection (5 days) and the turnover time of type II (sweet, bitter and umami) taste cells (8–10 days), we compared taste cell markers before and 10 days after TPF administration. The numbers of taste bud cells expressing gustducin (a bitter/sweet taste-related G-protein), T1R3 (a sweet/umami receptor component), PLCβ2 (a bitter/sweet/umami transduction molecule) and CaIV (a sour taste cell marker) were significantly decreased 10 days after TPF administration in mice that drank RT water. By contrast, the effects of TPF on the number of gustducin-, T1R3- and PLCβ2-expressing cells were suppressed by the drinking of ice-cold water (*p* < 0.05, one-way ANOVA and Tukey’s post-hoc test; Figure 4, Supplementary Table S4).

### 2.5. TPF-Induced Reductions in the Glossopharyngeal Nerve Responses to Taste Stimuli Were Suppressed by the Drinking of Ice-Cold Water

**Figure 5:**
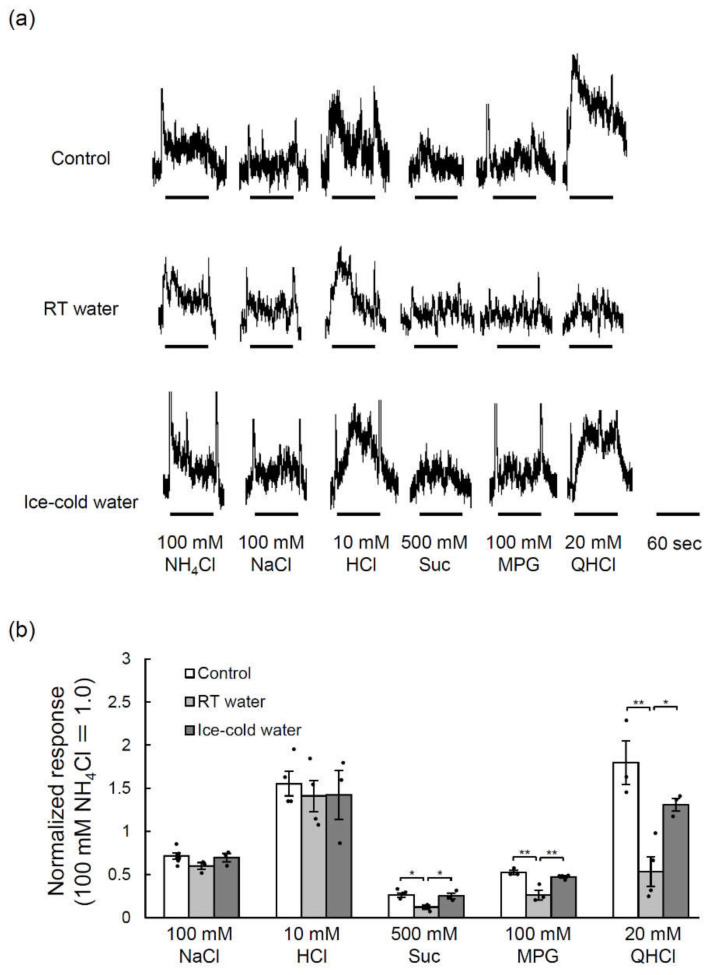
Glossopharyngeal nerve responses to various taste stimuli before and 10 days after the administration of TPF (docetaxel, cisplatin and 5-fluorouracil) in mice that drank room temperature (RT) water or ice-cold water. (**a**) Sample recordings of integrated whole nerve responses from the glossopharyngeal nerves before TPF administration (control; upper trace) and 10 days after TPF administration in mice that drank RT water (middle trace) or ice-cold water (lower trace). (**b**) Quantitation of the glossopharyngeal nerve responses to 100 mM NaCl, 10 mM HCl, 500 mM sucrose (Suc), 100 mM monopotassium glutamate (MPG) and 20 mM quinine hydrochloride (QHCl) before TPF administration and 10 days after TPF administration in mice that drank RT water or ice-cold water. The data are normalized to the response to 100 mM NH_4_Cl. Data are expressed as the mean ± SEM (*n* = 3–4 mice, each). Dots indicate individual data points. * *p* < 0.05, ** *p* < 0.01 (one-way ANOVA and Tukey-Kramer post-hoc test).

We further examined whether the taste responses of the glossopharyngeal nerve (which innervates the taste buds of the CVP) to various taste stimuli were changed by TPF administration and whether any such effects were inhibited by the drinking of ice-cold water. The glossopharyngeal nerve responses to sucrose (sweet taste), monopotassium glutamate (MPG, umami taste) and quinine hydrochloride (QHCl, bitter taste) were significantly smaller 10 days after TPF administration than before TPF administration (*p* < 0.05, one-way ANOVA and Tukey’s post-hoc test; Figure 5, Supplementary Table S4), although there were no clear effects on the responses to NaCl (*p* = 0.19) and HCl (*p* = 0.84) (one-way ANOVA, effect of mouse group; Figure 5, Supplementary Table S4). Moreover, the drinking of ice-cold water after TPF administration significantly suppressed the reduction of the glossopharyngeal nerve responses to sucrose, MPG and QHCl (*p* < 0.05, one-way ANOVA and Tukey’s post-hoc test; Figure 5, Supplementary Table S4).

## 3. Discussions

In this study, we found that the number of cells expressing various taste cell markers and the magnitudes of the responses of the glossopharyngeal nerve to tastants were significantly decreased in mice 10 days after the administration of TPF. These unfavorable effects of TPF on taste perception were suppressed in mice that drank ice-cold water. Taken together, our results suggest that anticancer drug-induced taste disorders may be due to a reduction in the number of taste receptor cells and that this can be partially prevented by cooling of the tongue with ice-cold water. We speculate that the effects of ice-cold water are mediated by a local narrowing of blood vessels that decreases the concentrations of anticancer drugs reaching the taste buds. We suggest that oral cryotherapy using an ice cube or ice cream might be a simple, low-cost and well-tolerated method of prophylaxis against anticancer drug-induced taste disorders in patients undergoing chemotherapy.

The clinical effectiveness of combination chemotherapy, such as the TPF regimen, has been confirmed in patients with unresectable squamous cell carcinoma of the head and neck [2,21]. Therefore, in the present study, mice administered a single intraperitoneal injection of TPF were used as an animal model of chemotherapy-induced taste dysfunction based on a clinically used regimen. According to the drug package inserts, the half-lives of docetaxel, cisplatin and 5-FU in humans are 20 h, 3 days and 20 min, respectively. Our observations that cell apoptosis and proliferation peaked at 5 days and that the number of taste cells was decreased at 10 days after the administration of a single dose of TPF are consistent with the longest half-life of 3 days for cisplatin.

Patients undergoing chemotherapy often experience taste disorders [6,8,22,23,24,25,26,27,28] that decrease QOL and cause anorexia that leads to a deterioration of nutritional status and a decline in ADL. The changes in taste perception may be due to cytotoxic damage to rapidly dividing taste bud cells [8]. Additionally, anticancer drugs can diffuse from the blood into the oral cavity via the saliva or gingival crevicular fluid to reach the taste receptor cells and produce a bitter taste [7]. Previous findings regarding the taste quality affected by chemotherapy have varied depending on the disease severity, treatment regimen, evaluation method and timing of data collection. For example, a cohort study reported that chemotherapy with 5-FU affected both sweet and salty taste sensitivities [29]. A self-report study identified disturbances in the perception of all taste types after the first chemotherapy cycle (frequency of occurrence: salty > sweet > bitter > sour) [30]. An investigation using taste strips found that all taste sensitivities (salty, sweet, bitter and sour) decreased during and after carboplatin-containing chemotherapy [31]. Another cohort study reported significant reductions in salty, sour and umami taste early in the chemotherapy cycle [32]. Thus, previously described alterations in taste after chemotherapy have usually occurred in more than one taste quality. The mouse glossopharyngeal nerve responses measured in the present study also demonstrated a tendency towards alternations in the responses to multiple tastants, particularly sucrose, MPG and QHCl (and possibly HCl and NaCl). Furthermore, the numbers of T1R3-, gustducin- and PLCβ2-positive type II cells (sweet, bitter and umami) and CaIV-positive type III cells (sour) were also reduced after TPF administration. These results appear to be consistent with those of human studies and suggest that our experimental system is a useful animal model of anticancer drug-induced taste disorders.

TUNEL and BrdU-based assays indicated that TPF injection resulted in significant increases in cell apoptosis from days 3–5 and cell proliferation from days 5–8. The proportion of TUNEL- and BrdU-positive cells on day 5 after TPF administration was ~2.6-times and ~4.8-times that of the control, respectively (from Tables S2 and S3). The above results suggest that there were relatively more apoptotic cells than proliferating cells on day 5 after TPF administration, as compared with the balance between apoptotic and proliferating cells before TPF administration. The increase in the number of proliferating cells may represent a recovery process involving an enhancement in the ability of stem cells or progenitor cells to proliferate and differentiate in response to cell apoptosis [33,34]. We also found that TPF administration led to a reduction in the number of cells expressing taste cell markers, which would be consistent with the observed enhancement of apoptosis. Notably, the extent of the decrease in the number of taste cells differed between the various taste cell types (~48% for gustducin-positive cells, ~34% for PLCβ2-positive cells, ~20% for CaIV-positive cells and ~16% for T1R3-positive cells; from Table S5). Since not all CaIV-positive cells are sour taste receptor cells [35,36], the extent of the decrease in cell number was likely much smaller for sour taste receptor cells than for the other taste cell types, and this would be consistent with the lack of an effect of TPF on the response of the glossopharyngeal nerve to sour taste. Our data raise the possibility that the fast turnover of type II cells and slow turnover of type III cells resulted in different rates of cell replacement after anticancer drug-induced injury.

It has been reported that a single dose of cyclophosphamide (a DNA-alkylating agent) exerts direct cytotoxic effects on taste bud cells in both the anterior and posterior regions of the mouse tongue [37,38], leading to decreases in the sensitivity to sucrose (sweet taste), number of fungiform taste papillae, expression of taste cell markers (PLCβ2 for type II and Synaptosomal-associated protein 25 [SNAP25] for type III) and expression of cell proliferation markers (Ki67 and BrdU). In addition, taste bud cells in the fungiform papillae appear to be more susceptible to cyclophosphamide (i.e., exhibit earlier changes) than those in the CVP [37,38]. In the present study, the administration of TPF affected multiple taste qualities and reduced the number of type II and type III cells. One possible explanation for our observations is that TPF targets taste stem/progenitor cells at the basal part of the taste buds where cell proliferation is frequently observed (Figure 3). Sonic hedgehog (Shh) is a key player in the formation and maintenance of taste buds, and its expression is preferentially restricted to the basal region of taste buds [39,40]. Vismodegib and sonidegib, which are antagonists of Shh that exert an anticancer effect via a different mechanism to TPF, directly alter Shh/smoothened (Smo)/glioma-associated oncogene homolog (Gli) signaling in the taste buds, resulting in a decrease in taste bud size, lower expression of taste cell markers (such as PLCβ2, T1R3 and gustducin), and a reduction in the responses of taste buds and taste nerves to five basic tastants [41,42]. Cells in the basal regions of the taste papillae in the posterior and anterior tongue also express leucine-rich repeat-containing G-protein coupled receptor (Lgr) 5 and 6, respectively. Lgr5-positive cells can generate all taste cell types found in adult taste buds including type I cells (supposedly salty taste-discriminating cells or supporting cells), type II cells (sweet, bitter and umami taste) and type III cells (sour taste), suggesting that Lgr5 is expressed in taste stem/progenitor cells in the posterior tongue [43]. The transcription factor, Skn-1a is crucial for regulating the binary differentiation of progenitor cells into type II cells and type III cells [44]. The results described above raise the possibility that the cells targeted by TPF may be Shh- or Lgr5-positive taste progenitor cells or more differentiated progenitor cells capable of dividing into type II and type III cells rather than cells that have already differentiated into mature taste cells under the regulation of Skn-1a.

Adverse effects of anticancer drugs are also observed in the gastrointestinal tract. For example, rats administered 150 mg/kg 5-FU as a single intraperitoneal injection exhibited an increase in the apoptosis index and a decrease in the mitosis index of all intestinal segments after 1 day that led to intestinal mucositis, shortening of villi, loss of normal crypt structure and inflammatory cell infiltration into the lamina propria after 3 days [45]. Another study in mice reported that 5-FU (23 mg/kg administered twice over 3 days) induced severe loss of colonic crypts and goblet cells (with no obvious effects on the epithelial brush border), acute intestinal inflammation and changes in the enteric nervous system that contributed to increased gastrointestinal transit [1]. The intestine is the fastest regenerating tissue in mammals with an average turnover of 3–5 days, and its regeneration is driven by Lgr5-expressing intestinal stem cells in the basal regions of the crypts [46]. Therefore, it is possible that anticancer drugs induce taste disorders through similar mechanisms to those underlying their effects in the gastrointestinal tract. The present study did not examine whether TPF caused inflammatory cell infiltration in the taste papillae or degeneration of taste neurons in the geniculate, petrosal and nodose ganglia. Therefore, further studies are needed to extend our findings.

Anticancer drug-induced taste disorders in patients often occur during the early phase of drug administration. Several studies have reported that cryotherapy reduces the severity of oral mucositis in patients undergoing chemotherapy [14,47,48], and the effect of oral cryotherapy was particularly apparent at 7–14 days after anticancer drug administration [47]. The Multinational Association of Supportive Care in Cancer guidelines recommend the use of cryotherapy for 30 min during the delivery of chemotherapy to reduce oral mucositis in patients receiving bolus doses of melphalan and 5-FU (https://www.mascc.org/). As a presumed protective mechanism, local cooling of the oral cavity with ice chips during chemotherapy is thought to cause reduced blood flow with vasoconstriction leading to decreased delivery of anticancer drugs to the oral mucosa as well as lowered metabolic activity. In fact, in vitro mucosa models incubated at lower temperatures, followed by an anticancer drug, showed higher cell viability and decreased cytokine production [49]. Oral cryotherapy was also reported to be effective for chemotherapy-induced hyperalgesia and neuropathy [50,51]. In the present study, the drinking of ice-cold water was associated with significant increases in the number of cells expressing taste cell markers (gustducin, T1R3 and PLCβ2) as well as enhancements in the taste nerve responses at 10 days after the administration of TPF. This recovery time appears to be consistent with the findings of the clinical trial performed by Katranci et al. [47]. Thus, oral cryotherapy using an ice cube might be a useful method for preventing or limiting anticancer drug-induced taste disorders in humans, although further clinical studies in patients undergoing chemotherapy would be required. In addition, chemotherapy entails at least several months in clinical practice. Most patients need to be treated with chemotherapy together with radiotherapy. Taste loss after irradiation is thought to be derived from continued natural taste cell death, paired with temporary interruption of cell replacement [34]. Although multiple factors are related to the cause of taste disorder in the side effects of cancer treatment, in this study, we focused on chemotherapy-induced taste disorder and found that oral cryotherapy might be effective. So, as a future study, it would be necessary to examine the long-term effect of oral cryotherapy on chemoradiotherapy for taste dysfunction patients.

## 4. Materials and Methods

### 4.1. Ethical Approval

All experimental procedures were performed in accordance with the Guide for the Care and Use of Laboratory Animals published by the National Institutes of Health and reviewed and approved by the Committee for Laboratory Animal Care and Use at Kyushu University, Japan (approval no. A30–276–0, 8 January 2019).

### 4.2. Animals

Adult male and female C57BL/6J (B6) mice (age, 8–16 weeks; weight, 20–30 g; Charles River, Tokyo, Japan) were used in all experiments. Mice were housed in groups of 2–5 animals and maintained at a constant room temperature of 24 ± 1 °C under a 12-h/12-h light/dark cycle (lights on at 0800). Food and water were available ad libitum. After TPF administration, mice were given free access to RT water (control group) or ice-cold water during the 12-h light cycle each day.

### 4.3. Solutions

TPF (docetaxel 20 mg/kg, cisplatin 6 mg/kg and 5-FU 17 mg/kg) was dissolved in saline (0.9% NaCl), and the drug concentrations were adjusted to provide the appropriate dose in a total volume of 1.0 mL. The concentration of TPF administered was based on the ratio and concentration reported previously [18]. The tastant solutions (100 mM NH_4_Cl, 100 mM NaCl, 10 mM HCl, 500 mM sucrose, 100 mM MPG and 20 mM QHCl) were prepared in distilled water. Reagents were purchased from Sanofi Co., Ltd. (Yamagata, Japan; docetaxel), Nichi-Iko Pharma Co., Ltd. (Toyama, Japan; cisplatin), Kyowa Kirin Co., Ltd. (Tokyo, Japan; 5-FU), Ajinomoto (Tokyo, Japan; IMP disodium salt) and Wako Pure Chemical Industries (Osaka, Japan; all other reagents).

### 4.4. Cell Proliferation Assay

The tongue of the mouse was resected 1 day after the intraperitoneal injection of BrdU (50 mg/kg body weight; Roche Diagnostics, Mannheim, Germany) [20]. The tongue was fixed with 4% paraformaldehyde in phosphate-buffered saline (PBS) for 45 min at 4 °C and then dehydrated with sucrose solutions (10% for 1 h, 20% for 1 h and 30% for 3 h, at 4 °C). A frozen block of tongue was embedded in optimal cutting temperature compound (Sakura Finetechnical, Tokyo, Japan) and sliced into serial sections (10-μm thickness) that were mounted on glass slides and air-dried. The sections were washed with PBS three times for 5 min and incubated with anti-BrdU monoclonal antibody according to the instructions for the use of the BrdU Labeling and Detection Kit II (Roche Diagnostics, Mannheim, Germany). After washing with PBS three times for 5 min, the sections were incubated with Alexa 647-conjugated anti-mouse IgG (Invitrogen, Carlsbad, CA, USA) and washed with PBS three times for 5 min. Images of taste cells expressing BrdU signals were obtained using an FV1000 confocal laser scanning microscope and Fluoview software (Olympus, Tokyo, Japan). Every fourth section was used in the analysis to avoid the double-counting of BrdU-positive cells.

### 4.5. Cell Death Assay

Sections of the CVP were prepared in a similar manner to that for the cell proliferation assay. The TUNEL assay was performed using the Click-iT^TM^ Plus TUNEL Assay (Invitrogen, Carlsbad, CA, USA). The sections were washed with Tris-NaCl-Tween (TNT) buffer three times for 5 min and incubated with TdT (terminal deoxynucleotidyl transferase) Reaction Buffer (Component A) for 10 min at 37 °C. After removal of the TdT reaction buffer, the sections were incubated for 60 min at 37 °C and then rinsed with distilled water. The sections were washed with 3% bovine serum albumin (BSA) and 0.1% Triton™ X-100 in PBS for 5 min, rinsed with PBS, and then incubated with the Click-iT™ Plus TUNEL reaction cocktail for 30 min at 37 °C in the dark. Then, the sections were washed with 3% BSA in PBS for 5 min and rinsed with PBS.

### 4.6. Labeling of Differentiated Cells

Differentiated type II and type III cells were identified using antibodies against gustducin, T1R3, PLCβ2 (type II cell markers) and CaIV (type III cell marker). Sections of the CVP were prepared in a similar manner to that for the cell proliferation assay. The sections were washed with TNT buffer three times for 5 min and preincubated with Blocking One-P solution (Nakalai Tesque, Kyoto, Japan) for 1 h at room temperature. Next, the sections were incubated with primary antibody against T1R3 (1:200; goat anti-T1R3, Santa Cruz Biotechnology, Dallas, TX, USA), gustducin (1:200; rabbit anti-Gα_gust_(I-20), Santa Cruz Biotechnology, Dallas, TX, USA), PLCβ2 (1:200; rabbit anti-PLCβ2, Santa Cruz Biotechnology, Dallas, TX, USA) or CaIV (1:100; goat anti-CA4, R&D Systems, Minneapolis, MN, USA) in Blocking One-P solution overnight at 4 °C. The sections were washed with TNT buffer three times for 5 min and then incubated for 2 h at room temperature with appropriate secondary antibody in 1% blocking reagent: Alexa Fluor 488 donkey anti-goat IgG (Invitrogen, Carlsbad, CA, USA) for T1R3, Alexa Fluor 594 donkey anti-rabbit IgG (Invitrogen, Carlsbad, CA, USA) for gustducin and PLCβ2, and Alexa Fluor 568 donkey anti-goat IgG (Invitrogen) for CaIV.

### 4.7. Image Analysis and Counting of Labeled Taste Cells

Images of cells expressing taste cell markers were obtained using an FV1000 confocal laser scanning microscope and Fluoview software (Olympus, Tokyo, Japan). To evaluate the number of labeled taste cells, Nomarski images were overlaid with immunofluorescence images. After confirming the outline of taste buds, sections were digitally zoomed to the area where individual taste cells could be easily distinguished. The number of positive cells displaying apparent apical processes and/or perinuclear region in each taste bud was counted in horizontal sections of CVP. Image-Pro Plus 4.0 (Media Cybernetics, Rockville, MD, USA) was used to exclude artifacts. A signal density greater than the mean plus two standard deviations of the negative control (primary antibody omitted) was considered a positive signal. The same taste cells showing positive signals in adjacent sections were counted only once. Cell counts were performed by observers blinded to conditions to avoid experimenter bias. We counted taste buds per CVP trench length and confirmed no significant difference in taste bud numbers across treatments (two-way ANOVA, *F*_4,95_ = 1.71, *p* > 0.05, effect of time, *F*_1,95_ = 0.15, *p* > 0.05, effect of drinking condition).

### 4.8. Statistical Analysis of the Data for Taste Cell Marker Expression

Comparisons of the data for taste cell marker expression were made using one-way ANOVA followed by the Tukey-Kramer post-hoc test and two-way ANOVA followed by the post-hoc *t*-test. IBM SPSS Statistics (IBM Corp., Armonk, NY, USA) was used to perform all calculations.

### 4.9. Nerve Recordings

Gustatory nerve responses to the lingual application of taste solutions were recorded from the glossopharyngeal nerve as described previously [52]. All procedures were performed under pentobarbital anesthesia (50–60 mg/kg body weight). The mouse was fixed in the supine position with its head in a holder to allow dissection of the glossopharyngeal nerve. After cannulation of the trachea, the digastric muscle and posterior horn of the hyoid bone were removed to expose the right glossopharyngeal nerve. Next, the glossopharyngeal nerve was dissected free from the underlying tissue and cut near its entrance to the jugular foramen. The whole nerve was placed on an electrode coated with Ag/AgCl. Neural activity was fed into an amplifier (K-1; Iyodenshikagaku, Nagoya, Japan) and monitored on an oscilloscope and audio monitor. Whole-nerve responses were integrated with a time constant of 1.0 s and recorded using a PowerLab system (PowerLab/sp4; ADInstruments, Bella Vista, NSW, Australia) connected to a computer. For taste stimulation of the CVP and foliate papillae, each side of the buccal region was incised from the corner of the mouth to just above the angle of the mandible. Slight tension was applied with a small suture sewn into the tip of the tongue to expose the papillae and open their trenches. Taste solutions (100 mM NH_4_Cl, 100 mM NaCl, 10 mM HCl, 500 mM sucrose, 100 mM MPG, or 20 mM QHCl) were perfused over the posterior tongue by gravity-driven flow for 60 s. The tongue was washed with distilled water for 60 s between successive stimulations. Only stable responses were used for the analyses.

### 4.10. Statistical Analysis of the Nerve Recording Data

The magnitudes of the integrated whole-nerve responses were measured from 5 to 55 s after the onset of stimulation, averaged and normalized to the response to 100 mM NH_4_Cl to reduce inter-mouse variations in the absolute responses. The relative responses were compared between groups (RT water vs. ice-cold water) using one-way ANOVA and the Tukey-Kramer post-hoc test.

## 5. Conclusions

The present study is the first to demonstrate that the drinking of ice-cold water suppressed the severity of anticancer drug-induced taste dysfunction in mice at the molecular and neural levels. The findings of this study raise the possibility that oral cryotherapy with an ice cube or ice cream might be a safe, low-cost and well-tolerated method of prophylaxis against anticancer drug-induced taste disorders in patients undergoing chemotherapy. However, randomized controlled clinical trials are needed to confirm whether oral cryotherapy might be an effective technique for reducing anticancer drug-induced taste disorders and improving the QOL and nutritional health of patients with cancer.

## Supplementary Materials

Supplementary materials can be found at https://www.mdpi.com/1422-0067/21/23/8958/s1.

## Abbreviations

5-FU	5-fluorouracil
ADL	Activities of daily living
BrdU	Bromodeoxyuridine
CaIV	Carbonic anhydrase IV
CVP	Circumvallate papillae
ENaC	Epithelial sodium channel
Gli	Glioma-associated oncogene homolog
GPCR	G protein-coupled receptor
Gust	Gα-gustducin
Lgr	Leucine-rich repeat-containing G-protein coupled receptor
MPG	Monopotassium glutamate
Otop1	Otopetrin-1
PBS	Phosphate-buffered saline
PLCβ2	Phospholipase C-beta 2
QHCl	Quinine hydrochloride
QOL	Quality of life
RT	Room temperature
Shh	Sonic hedgehog
Smo	Smoothened
SNAP25	Synaptosomal-associated protein 25
Suc	Sucrose
T1R	Taste receptor type 1 member
T2R	Taste receptor type 2
TNT	Tris-NaCl-Tween
TPF	Docetaxel, cisplatin and 5-fluorouracil
Trpm5	Transient receptor potential cation channel M5
TUNEL	Transferase-mediated dUTP nick end labeling
