# Supplementary material for: Drinking Ice-Cold Water Reduces the Severity of Anticancer Drug-Induced Taste Dysfunction in Mice
Source: Int J Mol Sci. 2020 Nov 25;21(23):8958. doi: 10.3390/ijms21238958 (PMC7728361; doi:10.3390/ijms21238958)
Supplement: Supplementary file 1 [file ijms-21-08958-s001.pdf]

International Journal of
*Molecular Sciences*

**Supplementary Table 1.** Results of ANOVA for weight, TUNEL signals and BrdU signals before and after TPF administration in mice that drank room temperature water or ice-cold water. The table is based on data shown in Figures 1–3.

| | One-way ANOVA | | Two-way ANOVA | | | | | |
|---|---|---|---|---|---|---|---|---|
| | | | Effect of drinking condition | | Effect of time | | Drinking condition × time interaction | |
| | ***F*** | ***P*** | ***F*** | ***P*** | ***F*** | ***P*** | ***F*** | ***P*** |
| Weight | | | | | | | | |
| (RT water) | (10, 33) = 2.23 | < 0.05 | (1, 60) = 0.34 | NS | (10, 60) = 12.26 | < 0.001 | (10, 60) = 2.20 | < 0.05 |
| (Ice-cold water) | (10, 33) = 3.78 | < 0.01 | | | | | | |
| TUNEL | | | | | | | | |
| (RT water) | (4, 54) = 8.00 | < 0.001 | (1, 115) = 11.62 | < 0.001 | (4, 115) = 12.09 | < 0.001 | (4, 115) = 1.99 | NS |
| (Ice-cold water) | (4, 59) = 4.20 | < 0.01 | | | | | | |
| BrdU | | | | | | | | |
| (RT water) | (4, 53) = 19.44 | < 0.001 | (1, 97) = 41.59 | < 0.001 | (4, 97) = 32.37 | < 0.001 | (4, 97) = 0.95 | NS |
| (Ice-cold water) | (4, 44) = 12.60 | < 0.001 | | | | | | |

Abbreviations: BrdU, bromodeoxyuridine; NS, not significant; RT, room temperature; TPF, docetaxel, cisplatin and fluorouracil; TUNEL, terminal deoxynucleotidyl transferase dUTP nick end labeling.

**Supplementary Table 2.** The number of TUNEL-positive cells per taste bud in the circumvallate papillae. The table is based on data shown in Figure 2.

| | **RT water** | **Ice-cold water** |
|---|---|---|
| Control | 0.15 | 0.13 |
| 3 days | 0.52 | 0.12 |
| 5 days | 0.72 | 0.49 |
| 8 days | 0.26 | 0.19 |
| 10 days | 0.26 | 0.20 |

Abbreviations: TUNEL, terminal deoxynucleotidyl transferase dUTP nick end labeling; RT, room temperature.

**Supplementary Table 3.** The number of BrdU-positive cells per taste bud in the circumvallate papillae. The table is based on data shown in Figure 3.

| | **RT water** | **Ice-cold water** |
|---|---|---|
| Control | 1.64 | 1.58 |
| 3 days | 2.42 | 2.00 |
| 5 days | 4.20 | 3.20 |
| 8 days | 3.03 | 2.40 |
| 10 days | 2.13 | 1.41 |

Abbreviations: BrdU, bromodeoxyuridine; RT, room temperature.

**Supplementary Table 4.** Results of ANOVA for taste cell marker expression (Gust, T1R3, PLCβ2 and CaIV) and glossopharyngeal nerve responses after TPF administration in mice that drank RT water or ice-cold water. The table is based on data shown in Figures 4–5.

| | **One-way ANOVA** | |
|---|---|---|
| | ***F*** | ***P*** |
| Gust | (2, 384) = 21.16 | < 0.001 |
| T1R3 | (2, 459) = 4.37 | < 0.05 |
| PLCβ2 | (2, 410) = 21.16 | < 0.001 |
| CaIV | (2, 387) = 3.67 | < 0.005 |
| NaCl | (2, 9) = 2.04 | 0.19 |
| HCl | (2, 8) = 0.17 | 0.84 |
| Sucrose | (2, 7) = 8.17 | < 0.05 |
| MPG | (2, 7) = 17.12 | < 0.01 |
| QHCl | (2, 7) = 13.13 | < 0.01 |

Abbreviations: CaIV, carbonic anhydrase IV; Gust, Gα-gustducin; MPG, monopotassium glutamate; PLCβ2, phospholipase C-beta 2; QHCl, quinine hydrochloride; RT, room temperature; T1R3, taste receptor type 1 member 3; TPF, docetaxel, cisplatin and fluorouracil.

**Supplementary Table 5.** The number of cells expressing taste cell markers in the circumvallate papillae. The table is based on data shown in Figure 4.

| | **Gust** | **T1R3** | **PLCβ2** | **CaIV** |
|---|---|---|---|---|
| Control | 6.28 | 4.06 | 6.08 | 2.82 |
| RT water (10 days) | 3.25 | 3.42 | 4.04 | 2.25 |
| Ice-cold water (10 days) | 4.08 | 3.99 | 4.63 | 2.56 |

Abbreviations: CaIV, carbonic anhydrase IV; Gust, Gα-gustducin; PLCβ2, phospholipase C-beta 2; RT, room temperature; T1R3, taste receptor type 1 member 3.
